# Triacylglycerol, fatty acid, and phytochemical profiles in a new red sorghum variety (Ji Liang No. 1) and its antioxidant and anti‐inflammatory properties

**DOI:** 10.1002/fsn3.886

**Published:** 2019-02-05

**Authors:** Yaqiong Zhang, Ming Li, Hang Gao, Bo Wang, Xu Tongcheng, Boyan Gao, Liangli (Lucy) Yu

**Affiliations:** ^1^ Beijing Advanced Innovation Center for Food Nutrition and Human Health Beijing Technology& Business University (BTBU) Beijing China; ^2^ Institute of Food and Nutraceutical Science School of Agriculture and Biology Shanghai Jiao Tong University Shanghai China; ^3^ Institute of Agro‐Food Science and Technology Shandong Provincial Key Laboratory of Agricultural Products Deep Processing Shandong Academy of Agricultural Science Jinan China; ^4^ Department of Nutrition and Food Science University of Maryland College Park Maryland

**Keywords:** anti‐inflammation, antioxidant, phenolic, red sorghum, triacylglycerol

## Abstract

In this study, a new red sorghum variety (Ji Liang No. 1) was investigated for its triacylglycerol (TAG) and fatty acid profiles, carotenoid and tocopherol compositions, total phenolic, total flavonoid and phenolic acid contents, and antioxidant and anti‐inflammatory properties. A total of 17 TAGs were identified in the red sorghum oil. Linoleic and oleic acids were the primary fatty acids, contributing more than 80% of the total fatty acids. β‐Carotene was the primary carotenoid at a level of 26.14 μg/g. α‐, γ‐, and δ‐tocopherols were at levels of 0.19, 4.08, and 0.10 μg/g, respectively. Moreover, acetone–water (60:40, v/v) extract of the red sorghum exhibited the greatest total phenolic content of 2.77 mg GAE/g and total flavonoid content of 5.44 mg RE/g. The extract had scavenging capacities against DPPH, ABTS ^+^, and peroxyl radicals and suppressed LPS stimulated IL‐1β, IL‐6, and COX‐2 mRNA expressions in a dose‐dependent manner. Ferulic, *p*‐coumaric, isoferulic, and *p*‐hydroxybenzoic acids were found in the red sorghum, with ferulic acid as the predominant phenolic acid and mostly in an insoluble bound form. These data indicated a potential utilization of the red sorghum in health‐promoting functional food or supplemental products.

## 1 INTRODUCTION

Sorghum (*Sorghum bicolor* L.) is the fifth most produced cereal crop after wheat, rice, maize, and barley in the world, and an important cereal used for human consumption in Asia, Africa, and other semi‐arid regions (de Morais Cardoso, Pinheiro, Martino, & Pinheiro‐Sant'Ana, 2015). Besides, sorghum is a good source of nutrients and multiple bioactive phytochemicals, mainly including procyanidins, flavonoids, phenolic acids, and other antioxidant compounds (Kaufman, Herald, Bean, Wilson, & Tuinstra, 2013; Shen, Zhang, Prinyawiwatkul, & Xu, 2013). These bioactive compounds have beneficial activities on human health. Some recent in vitro and in vivo studies have shown that the bioactive compounds isolated from sorghums benefit the gut microbiota and the parameters related to cancer, oxidative stress, inflammation, and many chronic metabolic diseases (Park, Lee, Chung, & Park, 2012; Shim, Kim, Jang, Ko, & Kim, 2013).

Fats and oils are important food ingredients, not only providing energy but also important for their nutritional values (Yang et al., 2018). Triacylglycerols (TAGs) are the major components and contribute to about 95% weights of fats and oils (Andrikopoulos, Chiou, & Mylona, 2004). Growing evidences show that the triacylglycerol and fatty acid compositions of fats and oils might significantly alter their flavor, stabilities, and physiological activities in food systems (Priore, Stanca, Gnoni, & Siculella, 2012; Yoshinaga et al., 2015). Some previous studies have shown that different sorghum varieties are additional vegetable oil sources due to the high concentrations of unsaturated fatty acids in their oil fractions (Mehmood, Orhan, Ahsan, Aslan, & Gulfraz, 2008; Pontieri et al., 2011). Meanwhile, carotenoids and tocopherols are also detected in sorghum oils, which are considered as two groups of well‐recognized natural antioxidants with potential health benefits (Cardoso et al., 2015; Chung, Yong, Lee, & Kim, 2013). However, to the best of our knowledge, the triacylglycerol composition in sorghum oil is still limited.

Besides, it is reported that sorghums with different genotypes and growing environments differed in their appearances, phytochemicals, physiological activities, and nutritional applications (Shen et al., 2018). Therefore, it is important to get such information for a new sorghum variety to determine whether it is a competitive source of the phytonutrients and potentially applied in functional foods or supplemental products. In the present study, a new high‐yielding red sorghum variety (Ji Liang No. 1) grown in saline‐alkali soil of Shandong was investigated for the profiles of TAGs, fatty acids, carotenoids, and tocopherols in its oil fraction. The sorghum variety was also examined for its phenolic acids composition, total phenolic, total flavonoid contents, and antioxidant and anti‐inflammatory capacities. The results from this study may be used to provide useful information for exploring the nutritional value of this red sorghum variety.

## 2 MATERIALS AND METHODS

### 2.1 Chemicals and reagents

2,2′‐azino‐bis (3‐ethylbenzothiazoline‐6‐sulfonic acid) diammonium salt (ABTS) (Catalog No. A1888), fluorescein (FL) (Catalog No. 32615), 2,2′‐azobis‐2‐aminopropane dihydrochloride (AAPH) (Catalog No. 440914), 6‐hydroxy‐2,5,7,8‐tetramethylchroman‐2‐carboxylic acid (Trolox) (Catalog No. 238813), 1,1‐diphenyl‐2‐picrylhydrazyl (DPPH) (Catalog No. D9132), β‐carotene (Catalog No. C4582), lutein (Catalog No. 07168), zeaxanthin (Catalog No. 14681), α‐ (Catalog No. 47783), δ‐ and γ‐tocopherols (Catalog No. 47784 and 47785), ferulic (Catalog No. PHR1791), isoferulic (Catalog No. 103012), *p*‐hydroxybenzoic (Catalog No. 240141), *p*‐coumaric (Catalog No. C9008), and gallic acids (Catalog No. G7384) were purchased from Sigma‐Aldrich (St. Louis, MO, USA). Rutin (Catalog No. R189033) was purchased from Aladdin reagent (Shanghai, China). Folin–Ciocalteu reagent (Catalog No. FB0467) was purchased from Macklin reagent (Shanghai, China). HPLC‐grade formic acid, acetonitrile, and methanol were obtained from Merck (Darmstadt, Germany). RAW 264.7 mouse macrophage was acquired from Chinese Academy of Sciences (Shanghai, China). Lipopolysaccharide (LPS) from *Escherichia coli 0111:B4* was obtained from Millipore (Billerica, MA, USA). iScript Advanced cDNA synthesis kit was purchased from Bio‐Rad (Hercules, CA, USA), while Dulbecco's Modified Eagle's Medium (DEME), fetal bovine serum (FBS), AB Power SYBR Green PCR Master Mix, TRIzol reagent and amphotericin B/streptomycin/penicillin were purchased from Life Technologies (Carlsbad, CA, USA). Water purified with a Millipore‐Q system was used for all experiments.

### 2.2 Red sorghum sample

The dehulled red sorghum sample (Ji Liang No. 1) was kindly donated by Shandong Academy of Agricultural Sciences (Jinan, Shandong, China) and ground into flour using a pulverizer (IKA A11 basic, Wilmington, NC, USA) to pass through a 40 mesh sieve. Then, the red sorghum flour was kept in a −20°C freezer in airtight containers until later use.

### 2.3 Extraction of red sorghum oil

About 800 mg of the red sorghum flour was extracted with approximately 100 ml of petroleum ether (boiling point of 30–60°C) for 4 hr via a Soxhlet apparatus. Then the petroleum ether was evaporated using a Buchi R215 rotary evaporator (Flawil, Switzerland) at a reduced pressure, and the remaining oil was weighed. The oil was stored at ambient temperature under nitrogen in the dark until further testing.

### 2.4 Chemical component analysis of red sorghum oil

#### 2.4.1 Triacylglycerols composition

The TAGs composition of red sorghum oil was identified using a Waters Xevo‐G2 Q‐TOF MS system combined with the Ultra Performance Convergence Chromatography (UPC^2^) system (Milford, MA, USA) according to a laboratory protocol (Gao et al., 2017).

#### 2.4.2 Fatty acid composition

Fatty acid methyl esters (FAMEs) were prepared from red sorghum oil by the saponification and boron trifluoride‐methanol method according to a laboratory protocol and subjected to GC‐MS (Loveland, CO, USA) analysis (Zhang et al., 2012).

#### 2.4.3 Carotenoid and tocopherol contents

The contents of β‐carotene, lutein, zeaxanthin, and α‐, δ‐, and γ‐tocopherols were determined simultaneously using a Waters Acuity UPLC coupled with SCIEX SelexION Triple Quad 5500 MS system according to a laboratory protocol (Slavin, Cheng, Luther, Kenworthy, & Yu, 2009).

### 2.5 Extraction of polyphenols from red sorghum

The single‐factor experiments were applied to optimize the extraction conditions on polyphenols from the defatted red sorghum. Four parameters, including extraction solvent (methanol, ethanol, and acetone), solvent concentration (40%, 60%, and 80%, v/v), solvent‐to‐solid ratio (10:1, 20:1, 30:1, and 40:1, ml/g), and ultrasonic time (0, 10, 20, 30, and 40 min) were investigated, respectively. The sonication water bath (KH‐500E, Kunshan, China) with fixed frequency at 40 kHz was used. The extract was kept in sealed containers in dark under nitrogen until further analysis.

### 2.6 Total phenolic contents

The red sorghum extract was analyzed for total phenolic contents (TPC) following a laboratory protocol (Moore, Liu, Zhou, & Yu, 2006). Briefly, 100 μl of sample was mixed with 500 μl of the Folin‐Ciocalteu reagent, 1.5 ml of 20 wt% sodium carbonate, and 6 ml of ultrapure water. After 2 hr of reaction, the absorbance at 765 nm was measured to calculate the TPC in samples using gallic acid as a standard. Results are expressed as milligrams of gallic acid equivalents per gram of dried red sorghum (mg GAE/g).

### 2.7 Total flavonoid contents

The red sorghum extract was analyzed for total flavonoid contents (TFC) following a previous procedure with some modifications (Frassinetti et al., 2018). Briefly, 250 μl of sample was mixed with 75 μl of 5 wt% sodium nitrite solution, 1 ml of ultrapure water and incubated at room temperature for 5 min. Then, 75 μl of 10 wt% aluminum nitrate solution was added and incubated for 6 min. Next, 500 μl of 1 M NaOH and 600 μl of ultrapure water were added. After 10 min of incubation, the absorbance at 510 nm was measured to calculate the TFC in samples using rutin as a standard. Results are expressed as milligrams of rutin equivalents per gram of dried red sorghum (mg RE/g).

### 2.8 Antioxidant activity assays

#### 2.8.1 DPPH˙ scavenging capacity

The red sorghum extract was evaluated for its DPPH˙ scavenging capacity according to a laboratory protocol (Whent, Slavin, Kenworthy, & Yu, 2010). Briefly, Trolox was used as a standard. 100 μl of sample, standard or blank, was added to 100 μl of freshly made DPPH˙ solution (0.2 mM) to initiate the reaction. The absorbance of the reaction mixtures was recorded at 517 nm for 30 min. DPPH˙ scavenging capacity was calculated from area under the curve and expressed as milligrams of Trolox equivalents (TE) per gram of dried red sorghum.

#### 2.8.2 ABTS˙^+^ scavenging capacity

Radical scavenging capacity of the red sorghum extract against ABTS˙^+^ was measured according to a laboratory protocol (Moore, Cheng, Su, & Yu, 2006). Briefly, ABTS˙^+^ working solution was prepared by reacting ABTS with manganese oxide and diluted to an absorbance of 0.7 at 734 nm. Trolox was used as a standard. The final reaction mixture consisted of 80 μl of sample, standard or blank, and 1 ml of ABTS˙^+^ working solution. The absorbance was read at 734 nm after 90 s of reaction. ABTS˙^+^ scavenging capacity was calculated using a standard curve prepared with Trolox and expressed as milligrams of TE per gram of dried red sorghum.

#### 2.8.3 Oxygen radical absorbance capacity

Oxygen radical absorbance capacity (ORAC) assay was conducted to assess peroxyl radical scavenging ability of the red sorghum extract according to a laboratory protocol (Slavin, Lu, Kaplan, & Yu, 2013). Briefly, Trolox was used as a standard. The initial reaction mixture consisted of 225 μl of freshly made 81.6 nM FL and 30 μl of sample, standard or blank. The mixtures were preheated in a 96‐well plate at 37°C for 10 min. Then, 25 μl of freshly made 0.36 M AAPH was added to each well to start the reaction. The fluorescence of assay mixture was recorded every 2 min over 2 hr at 37°C (*λ*_Ex_ = 485 nm and *λ*_Em_ = 528 nm). The results were calculated from area under the curve and expressed as milligrams of TE per gram of dried red sorghum.

### 2.9 Phenolic acid composition

The red sorghum was analyzed for its soluble free, soluble conjugated, insoluble bound and total (soluble free, soluble conjugated, and insoluble bound) phenolic acid compositions using a laboratory protocol (Moore et al., 2005). Acetone/methanol/water (7:7:6, v/v/v) was used to extract the soluble free and conjugated phenolic acids, whereas the insoluble bound phenolic acids in the solid residue were released by NaOH (2 M) hydrolysis. The free and conjugated phenolic acids in the acetone/methanol/water solution were separated based on their solubility in ethyl acetate‐ethyl ether (1:1, v/v) under acidic condition (pH 2). After evaporation of ethyl acetate and ethyl ether, the residue was re‐dissolved in methanol and filtered through a 0.22 μm membrane for UPLC‐Q‐TOF‐MS (Milford, MA, USA) analysis.

### 2.10 Anti‐inflammation effect of the red sorghum extract in RAW 264.7 mouse macrophage cells

RAW 264.7 mouse macrophage cells were cultured in DMEM containing 10% FBS and 1% amphotericin B/streptomycin/penicillin at 37°C under 5% CO_2_ in air to reach a confluence of 80%. The dried red sorghum extract re‐dissolved in pure DMSO was added into cell cultures at 10, 100, and 1,000 μg/ml for 24 hr. After pretreatment, LPS was added at an initial concentration of 10 ng/ml. After induction for 4 hr, culture media were discarded and cells were collected to perform RNA isolation and real‐time PCR according to a laboratory protocol (Niu et al., 2013). Briefly, cells were washed with 1× PBS and TRIzol reagent was added for total RNA isolation. iScriptTM Advanced cDNA Synthesis kit was used to reverse transcribe complementary DNA. Real‐time PCR was performed on ABI 7900HT Fast Real‐Time PCR System (Applied Biosystems, Carlsbad, CA, USA) using AB Power SYBR Green PCR Master Mix. Primers used in this study were as follows: IL‐1β (Forward: 5′‐GTTGACGGACCCCAAAAGAT‐3′, Reverse: 5′‐CCTCATCCTGGAAGGTCCAC‐3′); IL‐6 (Forward: 5′‐CACGGCCTTCCCTACTTCAC‐3′, Reverse: 5′‐TGCAAGTGCATCATCGTTGT‐3′); COX‐2 (Forward: 5′‐GGGAGTCTGGAACATTGTGAA‐3′, Reverse: 5′‐GCACGTTGATTGTAGGTGGACTGT‐3′). The mRNA amounts were normalized to an internal control, β‐actin mRNA (Forward: 5′‐GGAATGGGTCAGAAGGACTC‐3′, Reverse: 5′‐CATGTCGTCCCAGTTGGTAA‐3′). The following amplification parameters were used for PCR: 50°C for 2 min, 95°C for 2 min, and 40 cycles of amplification at 95°C for 15 s and 60°C for 15 s.

### 2.11 Statistical analysis

Data are reported as the mean ± standard deviation (SD) for triplicate determinations. One‐way ANOVA and Tukey's test were employed to identify differences in means. Statistics were analyzed using SPSS for Windows (version rel. 10.0.5; 1999, SPSS Inc., Chicago, IL, USA). Statistical significance was declared at *p *<* *0.05.

## 3 RESULTS AND DISCUSSION

### 3.1 Chemical component analysis of red sorghum oil

#### 3.1.1 Triacylglycerols composition

The red sorghum oil was first obtained by using a Soxhlet extraction method, and a crude oil content of 5–6 g per 100 g dried red sorghum was determined (data not shown). Then, the TAG profiles of red sorghum oil were identified by UPC^2^‐Q‐TOF MS. Based on the accurate elementary composition and MS^2^ fragmentation patterns provided by Q‐TOF MS, a total of 17 TAGs were detected and identified in red sorghum oil, and the relative concentration of each TAG was calculated using the peak normalization method and reported as g/100 g of TAGs (Table 1). The typical MS^1^ and MS^2^ spectra of TAGs were shown in Figure S1‐S3. Among the 17 identified TAGs, L‐L‐O, L‐L‐L, O‐O‐L, and L‐L‐P were the four major TAGs, with each contributing more than 10% of the total TAGs weight. Other six TAGs, including L‐O‐P, O‐O‐O, O‐O‐P, L‐L‐Ln, S‐O‐L, and Ln‐L‐P were also relatively abundant in red sorghum oil, ranging between 1.04% and 9.03% of the total TAGs weight. Moreover, some low content TAGs (<1% of the total TAGs weight), especially those containing arachidic (C20:0), eicosenoic acid (C20:1), and zoomaric (C16:1) acids were also identified, including L‐L‐Z, E‐O‐L, O‐O‐S, L‐L‐A, O‐O‐E, S‐S‐P, and L‐O‐A. To the best of our knowledge, the TAGs profile of red sorghum oil was reported for the first time.

**Table 1:** Identification and relative content of triacylglycerols (TAGs) in red sorghum oil^a^

Peak no.	RT (min)	Possible structure	Molecular formula	Mass ([M+NH_4_]^+^)	Calc. Mass ([M+NH_4_]^+^)	TAGs composition (g/100 g TAGs)
1	5.50	L‐L‐Z	C_55_H_96_O_6_	870.7452	870.7551	0.83 ± 0.04
2	5.77	L‐L‐Ln	C_57_H_96_O_6_	894.7532	894.7551	3.18 ± 0.05
3	5.84	Ln‐L‐P	C_55_H_96_O_6_	870.7542	870.7551	1.04 ± 0.03
4	5.95	L‐L‐L	C_57_H_98_O_6_	896.7695	896.7707	19.23 ± 0.21
5	6.05	L‐L‐P	C_55_H_98_O_6_	872.7712	872.7707	10.76 ± 0.16
6	6.26	L‐L‐O	C_57_H_100_O_6_	898.7871	898.7864	23.14 ± 0.38
7	6.40	L‐O‐P	C_55_H_100_O_6_	874.7864	874.7864	9.03 ± 0.15
8	6.64	O‐O‐L	C_57_H_102_O_6_	900.8024	900.802	17.96 ± 0.10
9	6.74	O‐O‐P	C_55_H_102_O_6_	876.8012	876.802	4.16 ± 0.10
10	7.02	O‐O‐O	C_57_H_104_O_6_	902.8163	902.8177	6.24 ± 0.12
11	7.40	S‐O‐L	C_57_H_104_O_6_	902.8175	902.8177	1.79 ± 0.04
12	7.75	E‐O‐L	C_59_H_106_O_6_	928.8325	928.8333	0.57 ± 0.01
13	7.88	O‐O‐S	C_57_H_106_O_6_	904.8327	904.8333	0.87 ± 0.03
14	8.13	L‐L‐A	C_59_H_106_O_6_	928.8352	928.8333	0.21 ± 0.03
15	8.2	O‐O‐E	C_59_H_108_O_6_	930.8495	930.849	0.23 ± 0.01
16	8.61	S‐S‐P	C_55_H_106_O_6_	880.8327	880.8333	0.45 ± 0.03
17	8.65	L‐O‐A	C_59_H_108_O_6_	930.8495	930.849	0.31 ± 0.01

*Notes*

RT: retention time; L: linoleic acid; O: oleic acid; P: palmitic acid; Ln: linolenic acid; S: stearic acid; Z: zoomaric acid; E: eicosenoic acid; A: arachidic acid.

a Values were analyzed in triplicates and reported as mean ± standard deviation (*SD*).

#### 3.1.2 Fatty acid composition

A total of six fatty acids were identified in red sorghum oil, including palmitic acid (C16:0), stearic acid (C18:0), oleic acid (C18:1), linoleic acid (C18:2), arachidic acid (C20:0), and eicosenoic acid (C20:1) (Table 2). Among them, linoleic acid was the predominant fatty acid (43.75%), followed by oleic acid (36.67%), palmitic acid (15.62%), stearic acid (2.94%), eicosenoic acid (0.22%), and arachidic acid (0.13%), which is consistent with previously published data on fatty acid composition of sorghum oils (Mehmood et al., 2008; Pontieri et al., 2011). There is considerable amount of linoleic and oleic acids in red sorghum oil, contributing more than 80% of the total fatty acids. These data indicated that the red sorghum oil may serve as a potential dietary source for unsaturated fatty acids. It has been recognized that unsaturated fatty acids are of great importance to human health, because they play an important role for the structure and function of biological membranes (Taylor, Schober, & Bean, 2006). Moreover, unsaturated fatty acids may lower the risk of thrombosis and related cardiovascular diseases due to their anti‐aggregating activity on blood lipoprotein particles (De Souza et al., 2015).

**Table 2:** Fatty acid compositions of red sorghum oil^a^

Fatty acid	C:D	Fatty acid composition (g/100 g fatty acids)
Palmitic acid	16:0	15.62 ± 0.27
Stearic acid	18:0	2.94 ± 0.06
Oleic acid	18:1	36.67 ± 0.60
Linoleic acid	18:2	43.75 ± 0.17
Arachidic acid	20:0	0.13 ± 0.04
Eicosenoic acid	20:1	0.22 ± 0.08

*Notes*

a Values were analyzed in triplicates and reported as mean ± standard deviation (*SD*). Fatty acids with lower concentration (<0.05%) are not listed above. “C” and “D” stands for Carbohydrate and Double bond, respectively; “C:D” is the ratio of the total amount of Carbon atoms of the fatty acid in relation to the number of double (unsaturated) bonds in it.

Besides, the GC‐MS analysis of fatty acid composition detected a total of six fatty acids, which are the same with the eight fatty acids from the UPC^2^‐Q‐TOF‐MS analysis of triacylglycerols composition. Only linolenic acid and zoomaric acid were not detected in fatty acid composition, which might be due to their relatively low contents.

#### 3.1.3 Carotenoid and tocopherol contents

The carotenoid composition including β‐carotene, lutein, and zeaxanthin was also examined for red sorghum oil (Table 3). The content of β‐carotene was 26.14 μg/g of red sorghum, which was found to be the primary carotenoid in red sorghum. Lutein was also present in red sorghum with its content of 5.37 μg/g of red sorghum, whereas no zeaxanthin was detected under the experimental conditions. Therefore, the total carotenoid contents were 31.51 μg/g of red sorghum, which was much greater than those of 3.82–19.5 μg/g in thirteen modern sorghum hybrids reported by Moreau, Harron, Powell, and Hoyt (2016). The difference in total carotenoid contents may be due to the different genotypes and cultivation locations of sorghum varieties.

**Table 3:** Carotenoid and tocopherol contents of red sorghum oil^a^

Sample	β‐Carotene (μg/g)	Lutein (μg/g)	Zeaxanthin (μg/g)	α‐Tocopherol (μg/g)	γ‐Tocopherol (μg/g)	δ‐Tocopherol (μg/g)
Red sorghum oil	26.14 ± 1.32	5.37 ± 0.09	nd	0.19 ± 0.00	4.08 ± 0.05	0.10 ± 0.01

*Notes*

a Values were analyzed in triplicates and reported as mean ± standard deviation (*SD*). nd represents not detectable.

Moreover, α‐, γ‐, and δ‐tocopherols were also detected in red sorghum oil, and their contents were ranked in the following order: γ‐tocopherol (4.08 μg/g) > α‐tocopherol (0.19 μg/g) > δ‐tocopherol (0.10 μg/g) (Table 3). γ‐tocopherol was found to be the most abundant tocopherol in red sorghum oil, which was in agreement with a previous study of sorghum breakfast cereal samples (Anunciação et al., 2017).

### 3.2 Optimization of extraction conditions for polyphenols in red sorghum

**Figure 1:**
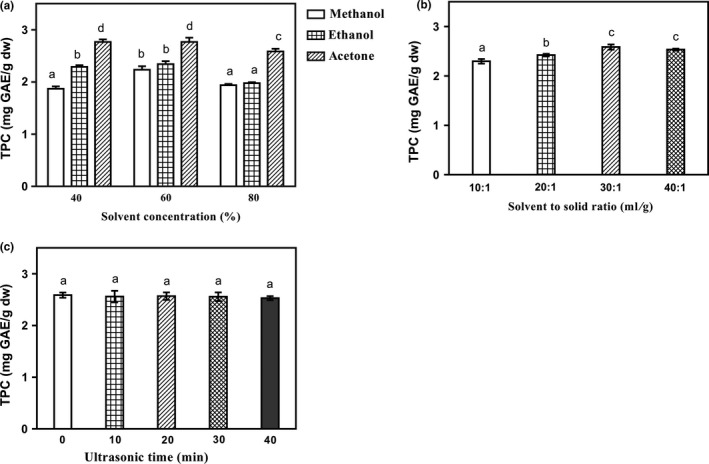
Effects of extraction solvent and solvent concentration (a), solvent‐to‐solid ratio (b) and ultrasonic time (c) on the yields of total phenolic contents (TPC) in single‐factor experiments. The vertical bars represent the standard deviation (SD) of each data point (*n* = 3). Different letters represent significant differences (*p *<* *0.05)

Single‐factor experiment was designed to evaluate the extraction solvent, solvent concentration, solvent‐to‐solid ratio, and ultrasonic time on the extraction yields of TPC from the defatted red sorghum. Generally, solvent mixtures containing methanol, ethanol or acetone combined with water in the concentration range of 40%–80% (v/v) were most commonly used to extract phenolic components from plant materials (Lv et al., 2013; Santos et al., 2018). Therefore, these three kinds of solvents with the concentration of 40%, 60%, and 80% (v/v) were studied, respectively. As shown in Figure 1a, the extraction yield for TPC significantly increased with the methanol concentration increasing from 40% to 60% (v/v) and peaked at 60% (v/v). Then it decreased with the methanol concentration further increasing to 80% (v/v) (*p *<* *0.05). The similar trend was also observed when using ethanol or acetone as the extraction solvent. Moreover, acetone with the concentration of 60% (v/v) had the greatest absolute value of TPC, when comparing with those of methanol or ethanol with the same concentration. This result may be explained by the similar polarity nature between acetone/water mixtures (60:40, v/v) and phenolic components in red sorghum, and therefore a higher solubility of phenolic components was achieved (Rostagno & Prado, 2013).

The solvent‐to‐solid ratio determined the concentration gradient of extraction medium and phenolic components during the extraction process. The higher the concentration gradient was, the faster mass transfer rate reached, and the more thorough the extraction gained when they reached the balance (Rostagno & Prado, 2013). As shown in Figure 1b, the extraction yield for TPC increased significantly (*p *<* *0.05), when the solvent‐to‐solid ratio increased from 10:1 to 30:1 with the extraction medium of acetone/water mixture (60:40, v/v) fixed. With further increase of solvent‐to‐solid ratio from 30:1 to 40:1, the TPC remained statistically unchanged (*p *>* *0.05). The similar tendency for this factor was also showed for the extraction of polyphenolic compounds from red sorghum bran (Luo et al., 2018).

Compared with the conventional extraction method, ultrasonic‐assisted extraction has been reported to have an enhanced extraction yield for phenolic compounds (Wang, Boussetta, Lebovka, & Vorobiev, 2018). In this study, the effect of ultrasonic time on the extraction yield of TPC was also investigated from 0 to 40 min with the extraction medium of acetone/water mixture (60:40, v/v) and solvent‐to‐solid ratio of 30:1 fixed (Figure 1c). However, the result indicated that the extraction yield of TPC was not significantly influenced by the ultrasonic time (*p *>* *0.05), which might due to that the soluble phenolic components had already been extracted completely without ultrasonic assistance. Moreover, in most cases, ultrasound is insufficient to break ester or ether bonds and releases some insoluble bound phenolic components, such as insoluble bound phenolic acid, despite the likelihood that the shearing force generated during cavitation would enhance mass transfer by increasing solid solubility and diffusion of solubilized phenolic components into the solvent (Đurović et al., 2018).

Based on the single‐factor experiment results, the optimized extraction conditions were determined as follows: the red sorghum was extracted with acetone/water mixture (60:40, v/v) at the solvent‐to‐solid ratio of 30:1. Under this extraction condition, the values of TPC and TFC of red sorghum extract were 2.77 mg GAE/g and 5.44 mg RE/g of red sorghum, respectively (Table 4).

**Table 4:** Total phenolic content (TPC), total flavonoid content (TFC), and free radical scavenging capacities of red sorghum extract^a^

Sample	TPC (mg GAE/g)	TFC (mg RE/g)	DPPH (mg Trolox/g)	ABTS (mg Trolox/g)	ORAC (mg Trolox/g)
Red sorghum extract	2.77 ± 0.07	5.44 ± 0.06	1.97 ± 0.05	13.71 ± 0.13	40.59 ± 0.32

*Notes*

ORAC: oxygen radical absorbance capacity.

Values were analyzed in triplicates and reported as mean ± standard deviation (*SD*). GAE and RE represent gallic acid and rutin equivalents, respectively.

### 3.3 Antioxidant activity of red sorghum extract

In this study, DPPH radical, ABTS radical cation scavenging capacities, and ORAC assays were chosen by evaluating the antioxidant activity of red sorghum extract, respectively. As shown in Table 4, DPPH, ABTS, and ORAC values of the red sorghum extract were measured to be 1.97, 13.71, and 40.59 mg TE/g of red sorghum, respectively. It seemed that the ABTS value was 6–7 times higher than the DPPH value. It is known that anthocyanins are the major extractable phenols from red or black sorghums (Akogou, Kayodé, den Besten, & Linnemann, 2017), and they contribute a major portion of the measured antioxidant activity of sorghum samples. Since anthocyanins absorb maximally at 475–485 nm, the color interference with the DPPH chromogen (absorbed maximally at 515 nm) may occur and result in the relatively lower DPPH value. Arnao's work also reported the similar color interference phenomenon (Arnao, 2000). Moreover, compared with the DPPH or ABTS values, the red sorghum extract showed the greatest ORAC value of 40.59 mg TE/g of red sorghum, which indicated that phenolic compounds in red sorghum extract were more efficient at quenching peroxyl radical than DPPH and ABTS cation radicals. This result was consistent with our previous observations for soybean extracts (Slavin et al., 2013; Whent et al., 2010).

### 3.4 Phenolic acid compositions

As reported in our previous studies, phenolic acids existed in a variety of forms in grains, including soluble free, soluble conjugated, and insoluble bound form (Moore, Luther, Cheng, & Yu, 2009). Therefore, soluble free, soluble conjugated, and insoluble bound phenolic acid compositions in red sorghum extract were also determined, respectively. As a result, ferulic, *p*‐coumaric, isoferulic, and *p*‐hydroxybenzoic acids were detected under the experimental conditions (Table 5). Similar to other sorghum varieties (Shen et al., 2018) and cereal samples (Moore et al., 2005), the total content of ferulic acid in red sorghum showed the highest level compared to other detected phenolic acids, accounting for 128.93 μg/g of red sorghum and over 47% of the total identified phenolic acids on a weight basis. Most of the ferulic acid in red sorghum was insoluble bound with the content of 88.49 μg/g of red sorghum, and this level accounted for 68.6% of the total ferulic acid. Also, each gram of red sorghum contained 11.21 μg of soluble free, 39.56 μg of soluble conjugated, 29.64 μg of insoluble bound *p*‐coumaric acid, which was the second high content of phenolic acid in red sorghum. Actually, ferulic and *p*‐coumaric acids are also the most commonly detected phenolic acids for sorghum samples according to some previous publications (Chiremba, Taylor, Rooney, & Beta, 2012; Luthria & Liu, 2013), which may be due to their relative high contents. Moreover, isoferulic and *p*‐hydroxybenzoic acids were also detected in red sorghum with their total contents ranging from 45 to 49 μg/g of red sorghum. For the isoferulic acid, the insoluble bound was the primary phenolic acid form, accounting for 79.2% of the total identified isoferulic acid on a weight basis, whereas no soluble free isoferulic acid was detected. *p*‐hydroxybenzoic acid was the only detected phenolic acid with approximate content of soluble conjugated and insoluble bound forms, but only less content of soluble free *p*‐hydroxybenzoic acid was detected. To the best of our knowledge, this is the first time that isoferulic acid is detected in the sorghum sample. Interestingly, none of caffeic, sinapic, and protocatechuic acids were detected in this study, which were reported in some sorghum grain varieties previously (Wu, Johnson, Bornman, Bennett, & Fang, 2017; Wu et al., 2016). The difference in phenolic acid compositions from this and others’ studies might partially be explained by the different sorghum varieties, as well as different preparation and detection conditions of samples. It was also possible that the growing seasons and locations might result in the different phenolic acid compositions in different studies.

**Table 5:** Phenolic acid composition of red sorghum extract^a^

Phenolic acids	Soluble free (μg/g)	Soluble conjugated (μg/g)	Insoluble bound (μg/g)	Total phenolic acids (μg/g)
Ferulic acid	4.62 ± 0.66	35.82 ± 1.20	88.49 ± 5.89	128.93 ± 7.75
*p*‐coumaric acid	11.21 ± 1.40	39.56 ± 1.18	29.64 ± 0.89	80.40 ± 3.47
Isoferulic acid	nd	10.18 ± 1.18	38.77 ± 0.67	48.95 ± 1.85
*p*‐hydroxybenzoic acid	3.41 ± 0.43	20.31 ± 2.11	21.54 ± 0.55	45.26 ± 3.09

*Notes*

a Values were analyzed in triplicates and reported as mean ± standard deviation (*SD*). nd represents not detectable.

### 3.5 Anti‐inflammatory effect of red sorghum extract in RAW 264.7 mouse macrophage cells

Chronic inflammation has been recognized as a risk factor for a number of health problems, including cardiovascular disease, cancer, and obesity (Väyrynen et al., 2018). Several cytokines, including interleukin‐1β (IL‐1β), IL‐6, and cyclo‐oxygenase (COX‐2), are three critical proinflammation mediators involved in multiple inflammatory pathways. The inhibition of their mRNA expression may lead to alleviation of the inflammatory responses. In this study, the effect of red sorghum extract on IL‐1β, IL‐6, and COX‐2 mRNA expressions was examined for their potential anti‐inflammatory activities.

**Figure 2:**
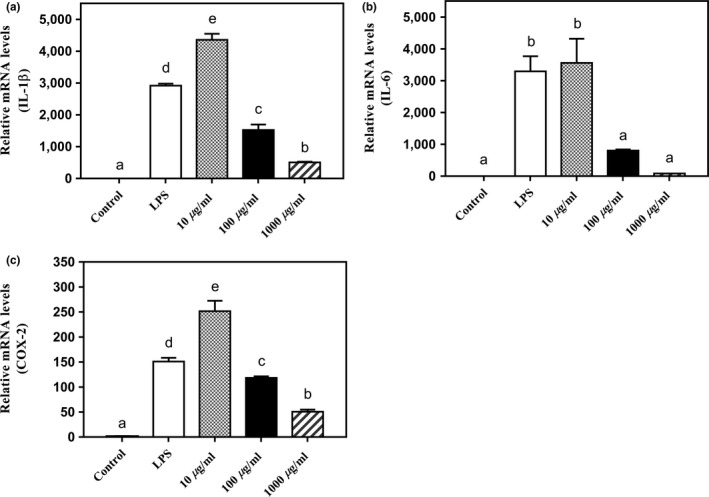
Effects of red sorghum extract on (a) IL‐1β, (b) IL‐6, and (c) COX‐2 mRNA expressions in mouse RAW 264.7 macrophage cells. Control and LPS contained the same concentration of DMSO as the treatment samples. LPS stands for lipopolysaccharide. The vertical bars represent the standard deviation (SD) of each data point (*n* = 3). Different letters represent significant differences (*p *<* *0.05). LPS: lipopolysaccharide

As shown in Figure 2, the red sorghum extract significantly suppressed the LPS‐induced IL‐1β, IL‐6, and COX‐2 mRNA expressions at 100 or 1,000 μg/ml initial treatment concentration (*p *<* *0.05), whereas 10 μg/ml of red sorghum extract did not have significant inhibitory effect. Moreover, at 100 or 1,000 μg/ml initial treatment concentration, the inhibitory effect of red sorghum extract was in a dose‐dependent way. The higher initial treatment concentration of red sorghum extract was, the stronger inhibitory effect was observed. In this study, the red sorghum extract showed the strongest inhibitory effect in suppressing IL‐6 mRNA expression at 1,000 μg/ml initial treatment concentration, which inhibited 97.5% of IL‐6 mRNA expression under the experimental conditions (Figure 2b). Taken together, these results indicated that the red sorghum extract might have an anti‐inflammatory potential.

## 4 CONCLUSION

In summary, the present study demonstrated the triacylglycerol composition of red sorghum oil for the first time and indicated that the new red sorghum variety may serve as a potential dietary source for unsaturated fatty acids and other beneficial phytochemicals, such as tocopherols, carotenoids, and phenolic compounds, therefore contributing to its antioxidant and anti‐inflammatory properties. Additional studies with larger sample sizes and detailed growing conditions are necessary to further investigate the relationship between chemical compositions of red sorghum and its health‐promoting or disease‐preventing activities in humans.

## CONFLICT OF INTEREST

The authors declare that there are no conflict of interests.

## ETHICAL STATEMENT

This article does not involve any human or animal studies.

## References

[fsn3886-bib-0001] Akogou, F. U. G. , Kayodé, A. P. P. , den Besten, H. M. W. , & Linnemann, A. R. (2017). Extraction methods and food uses of a natural red colorant from dye sorghum. Journal of the Science of Food and Agriculture, 98, 361–368. 28600852 10.1002/jsfa.8479

[fsn3886-bib-0002] Andrikopoulos, N. K. , Chiou, A. , & Mylona, A. (2004). Triacylglycerol species of less common edible vegetable oils. Food Reviews International, 20(4), 389–405. 10.1081/FRI-200033470

[fsn3886-bib-0003] Anunciação, P. C. , Cardoso, L. D. M. , Gomes, J. V. P. , Della Lucia, C. M. , Carvalho, C. W. P. , Galdeano, M. C. , … Pinheiro‐Sant'Ana, H. M. (2017). Comparing sorghum and wheat whole grain breakfast cereals: Sensorial acceptance and bioactive compound content. Food Chemistry, 221, 984–989. 10.1016/j.foodchem.2016.11.065 27979303

[fsn3886-bib-0004] Arnao, M. B. (2000). Some methodological problems in the determination of antioxidant activity using chromogen radicals: A practical case. Trends in Food Science and Technology, 11(11), 419–421. 10.1016/S0924-2244(01)00027-9

[fsn3886-bib-0005] Cardoso, L. D. M. , Pinheiro, S. S. , Da Silva, L. L. , De Menezes, C. B. , De Carvalho, C. W. P. , Tardin, F. D. , … Pinheiro‐Sant'Ana, H. M. (2015). Tocochromanols and carotenoids in sorghum (*Sorghum bicolor* L.): Diversity and stability to the heat treatment. Food Chemistry, 172, 900–908. 10.1016/j.foodchem.2014.09.117 25442636

[fsn3886-bib-0006] Chiremba, C. , Taylor, J. R. N. , Rooney, L. W. , & Beta, T. (2012). Phenolic acid content of sorghum and maize cultivars varying in hardness. Food Chemistry, 134(1), 81–88. 10.1016/j.foodchem.2012.02.067 22500656

[fsn3886-bib-0007] Chung, I. M. , Yong, S. J. , Lee, J. , & Kim, S. H. (2013). Effect of genotype and cultivation location on β‐sitosterol and α‐, β‐, γ‐, and δ‐tocopherols in sorghum. Food Research International, 51(2), 971–976. 10.1016/j.foodres.2013.02.027

[fsn3886-bib-0008] de Morais Cardoso, L. , Pinheiro, S. S. , Martino, H. S. D. , & Pinheiro‐Sant'Ana, H. M. (2015). Sorghum (*Sorghum bicolor* L.): Nutrients, bioactive compounds, and potential impact on human health. Critical Reviews in Food Science and Nutrition, 57(2), 372–390. 10.1080/10408398.2014.887057 25875451

[fsn3886-bib-0009] De Souza, R. J. , Mente, A. , Maroleanu, A. , Cozma, A. I. , Ha, V. , Kishibe, T. , … Anand, S. S. (2015). Intake of saturated and trans unsaturated fatty acids and risk of all cause mortality, cardiovascular disease, and type 2 diabetes: Systematic review and meta‐analysis of observational studies. BMJ, 351, h3978 10.1136/bmj.h3978 26268692 PMC4532752

[fsn3886-bib-0010] Đurović, S. , Nikolić, B. , Luković, N. , Jovanović, J. , Stefanović, A. , Šekuljica, N. , … Knežević‐Jugović, Z. (2018). The impact of high‐power ultrasound and microwave on the phenolic acid profile and antioxidant activity of the extract from yellow soybean seeds. Industrial Crops and Products, 122, 223–231.

[fsn3886-bib-0011] Frassinetti, S. , Moccia, E. , Caltavuturo, L. , Gabriele, M. , Longo, V. , Bellani, L. , … Giorgetti, L. (2018). Nutraceutical potential of hemp (*Cannabis sativa* L.) seeds and sprouts. Food Chemistry, 262, 56–66. 10.1016/j.foodchem.2018.04.078 29751921

[fsn3886-bib-0012] Gao, B. Y. , Luo, Y. H. , Lu, W. Y. , Liu, J. , Zhang, Y. Q. , & Yu, L. L. (2017). Triacylglycerol compositions of sunflower, corn and soybean oils examined with supercritical CO_2_ ultra‐performance convergence chromatography combined with quadrupole time‐of‐flight mass spectrometry. Food Chemistry, 218, 569–574. 10.1016/j.foodchem.2016.09.099 27719951

[fsn3886-bib-0013] Kaufman, R. C. , Herald, T. J. , Bean, S. R. , Wilson, J. D. , & Tuinstra, M. R. (2013). Variability in tannin content, chemistry and activity in a diverse group of tannin containing sorghum cultivars. Journal of the Science of Food and Agriculture, 93(5), 1233–1241. 10.1002/jsfa.5890 23011944

[fsn3886-bib-0014] Luo, X. P. , Cui, J. M. , Zhang, H. H. , Duan, Y. Q. , Zhang, D. , Cai, M. H. , & Chen, G. Y. (2018). Ultrasound assisted extraction of polyphenolic compounds from red sorghum (*Sorghum bicolor* L.) bran and their biological activities and polyphenolic compositions. Industrial Crops and Products, 112, 296–304. 10.1016/j.indcrop.2017.12.019

[fsn3886-bib-0015] Luthria, D. L. , & Liu, K. (2013). Localization of phenolic acids and antioxidant activity in sorghum kernels. Journal of Functional Foods, 5(4), 1751–1760. 10.1016/j.jff.2013.08.001

[fsn3886-bib-0016] Lv, J. L. , Lu, Y. J. , Niu, Y. G. , Whent, M. , Ramadan, M. F. , Costa, J. , & Yu, L. L. (2013). Effect of genotype, environment, and their interaction on phytochemical compositions and antioxidant properties of soft winter wheat flour. Food Chemistry, 138(1), 454–462. 10.1016/j.foodchem.2012.10.069 23265511

[fsn3886-bib-0017] Mehmood, S. , Orhan, I. , Ahsan, Z. , Aslan, S. , & Gulfraz, M. (2008). Fatty acid composition of seed oil of different sorghum bicolor varieties. Food Chemistry, 109(4), 855–859. 10.1016/j.foodchem.2008.01.014 26050001

[fsn3886-bib-0018] Moore, J. , Cheng, Z. H. , Su, L. , & Yu, L. L. (2006). Effects of solid‐state enzymatic treatments on the antioxidant properties of wheat bran. Journal of Agricultural and Food Chemistry, 54(24), 9032–9045. 10.1021/jf0616715 17117788

[fsn3886-bib-0019] Moore, J. , Hao, Z. G. , Zhou, K. Q. , Luther, M. , Costa, J. , & Yu, L. L. (2005). Carotenoid, tocopherol, phenolic acid, and antioxidant properties of maryland‐grown soft wheat. Journal of Agricultural and Food Chemistry, 53(17), 6649–6657. 10.1021/jf050481b 16104780

[fsn3886-bib-0020] Moore, J. , Liu, J. G. , Zhou, K. Q. , & Yu, L. L. (2006). Effects of genotype and environment on the antioxidant properties of hard winter wheat bran. Journal of Agricultural and Food Chemistry, 54(15), 5313–5322. 10.1021/jf060381l 16848511

[fsn3886-bib-0021] Moore, J. , Luther, M. , Cheng, Z. H. , & Yu, L. L. (2009). Effects of baking conditions, dough fermentation, and bran particle size on antioxidant properties of whole‐wheat pizza crusts. Journal of Agricultural and Food Chemistry, 57(3), 832–839. 10.1021/jf802083x 19138086

[fsn3886-bib-0022] Moreau, R. A. , Harron, A. F. , Powell, M. J. , & Hoyt, J. L. (2016). A comparison of the levels of oil, carotenoids, and lipolytic enzyme activities in modern lines and hybrids of grain sorghum. Journal of the American Oil Chemists Society, 93(4), 569–573. 10.1007/s11746-016-2799-4

[fsn3886-bib-0023] Niu, Y. G. , Gao, B. Y. , Slavin, M. , Zhang, X. W. , Yang, F. , Bao, J. S. , … Yu, L. L. (2013). Phytochemical compositions, and antioxidant and anti‐inflammatory properties of twenty‐two red rice samples grown in Zhejiang. LWT – Food Science and Technology, 54(2), 521–527. 10.1016/j.lwt.2013.06.018

[fsn3886-bib-0024] Park, J. H. , Lee, S. H. , Chung, I. M. , & Park, Y. (2012). Sorghum extract exerts an anti‐diabetic effect by improving insulin sensitivity via ppar‐γ in mice fed a high‐fat diet. Nutrition Research and Practice, 6(4), 322–327. 10.4162/nrp.2012.6.4.322 22977686 PMC3439576

[fsn3886-bib-0025] Pontieri, P. , di Fiore, R. , Troisi, J. , Bean, S. R. , Roemer, E. , Okot, J. , … Massardo, D. R. (2011). Chemical composition and fatty acid content of white food sorghums grown in different environments. Maydica, 56(1), 51–58.

[fsn3886-bib-0026] Priore, P. , Stanca, E. , Gnoni, G. V. , & Siculella, L. (2012). Dietary fat types differently modulate the activity and expression of mitochondrial carnitine/acylcarnitine translocase in rat liver. Biochimica et Biophysica Acta, 1821(10), 1341–1349. 10.1016/j.bbalip.2012.07.008 22819991

[fsn3886-bib-0027] Rostagno, M. , & Prado, J. (2013). Natural product extraction: Principles and applications. London, UK: RSC Press 10.1039/1757-7047

[fsn3886-bib-0028] Santos, C. H. K. , Baqueta, M. R. , Coqueiro, A. , Dias, M. I. , Barros, L. , Barreiro, M. F. , … Leimann, F. V. (2018). Systematic study on the extraction of antioxidants from pinhão, (*Araucaria angustifolia*, (bertol.) kuntze) coat. Food Chemistry, 261, 216–223. 10.1016/j.foodchem.2018.04.057 29739586

[fsn3886-bib-0029] Shen, S. Y. , Huang, R. , Li, C. , Wu, W. Y. , Chen, H. L. , Shi, J. , … Ye, X. Q. (2018). Phenolic compositions and antioxidant activities differ significantly among sorghum grains with different applications. Molecules, 23(5), 1203–1217. 10.3390/molecules23051203 PMC6100422 29772811

[fsn3886-bib-0030] Shen, Y. , Zhang, X. , Prinyawiwatkul, W. , & Xu, Z. (2013). Phytochemicals in sweet sorghum (dura) and their antioxidant capabilities against lipid oxidation. Journal of Agricultural and Food Chemistry, 61(51), 12620–12624. 10.1021/jf4040157 24295015

[fsn3886-bib-0031] Shim, T. J. , Kim, T. M. , Jang, K. C. , Ko, J. Y. , & Kim, D. J. (2013). Toxicological evaluation and anti‐inflammatory activity of a golden gelatinous sorghum bran extract. Journal of the Agricultural Chemical Society of Japan, 77(4), 697–705. 10.1271/bbb.120731 23563532

[fsn3886-bib-0032] Slavin, M. , Cheng, Z. H. , Luther, M. , Kenworthy, W. , & Yu, L. L. (2009). Antioxidant properties and phenolic, isoflavone, tocopherol and carotenoid composition of Maryland‐grown soybean lines with altered fatty acid profiles. Food Chemistry, 114(1), 20–27. 10.1016/j.foodchem.2008.09.007

[fsn3886-bib-0033] Slavin, M. , Lu, Y. J. , Kaplan, N. , & Yu, L. L. (2013). Effects of baking on cyanidin‐3‐glucoside content and antioxidant properties of black and yellow soybean crackers. Food Chemistry, 141(2), 1166–1174. 10.1016/j.foodchem.2013.04.039 23790899

[fsn3886-bib-0034] Taylor, J. R. N. , Schober, T. J. , & Bean, S. R. (2006). Novel food and non‐food uses for sorghum and millets. Journal of Cereal Science, 44(3), 252–271. 10.1016/j.jcs.2006.06.009

[fsn3886-bib-0035] Väyrynen, J. P. , Tuomisto, A. , Väyrynen, S. A. , Klintrup, K. , Karhu, T. , Mäkelä, J. , … Mäkinen, M. J. (2018). Preoperative anemia in colorectal cancer: Relationships with tumor characteristics, systemic inflammation, and survival. Scientific Reports, 8(1), 1126 10.1038/s41598-018-19572-y 29348549 PMC5773501

[fsn3886-bib-0036] Wang, L. , Boussetta, N. , Lebovka, N. , & Vorobiev, E. (2018). Selectivity of ultrasound‐assisted aqueous extraction of valuable compounds from flesh and peel of apple tissues. LWT – Food Science and Technology, 93, 511–516. 10.1016/j.lwt.2018.04.007

[fsn3886-bib-0037] Whent, M. , Slavin, M. , Kenworthy, W. , & Yu, L. L. (2010). Potential relationships between fatty acid compositions and phytochemicals of selected low linolenic soybeans grown in Maryland. Journal of the American Oil Chemists’ Society, 87(5), 549–558. 10.1007/s11746-009-1519-8

[fsn3886-bib-0038] Wu, G. , Johnson, S. K. , Bornman, J. F. , Bennett, S. J. , Clarke, M. W. , Singh, V. , & Fang, Z. (2016). Growth temperature and genotype both play important roles in sorghum grain phenolic composition. Scientific Reports, 6, 21835 10.1038/srep21835 26907726 PMC4764825

[fsn3886-bib-0039] Wu, G. , Johnson, S. K. , Bornman, J. F. , Bennett, S. J. , & Fang, Z. (2017). Changes in whole grain polyphenols and antioxidant activity of six sorghum genotypes under different irrigation treatments. Food Chemistry, 214, 199–207. 10.1016/j.foodchem.2016.07.089 27507466

[fsn3886-bib-0040] Yang, R. N. , Zhang, L. X. , Li, P. W. , Yu, L. , Mao, J. , Wang, X. P. , & Zhang, Q. (2018). A review of chemical composition and nutritional properties of minor vegetable oils in China. Trends in Food Science and Technology, 74, 26–32. 10.1016/j.tifs.2018.01.013

[fsn3886-bib-0041] Yoshinaga, K. , Sasaki, K. , Watanabe, H. , Nagao, K. , Inoue, N. , Shirouchi, B. , … Gotoh, N. (2015). Differential effects of triacylglycerol positional isomers containing n‐3 series highly unsaturated fatty acids on lipid metabolism in C57BL/6jJmice. The Journal of Nutritional Biochemistry, 26(1), 57–63. 10.1016/j.jnutbio.2014.09.004 25448607

[fsn3886-bib-0042] Zhang, X. W. , Gao, B. Y. , Shi, H. M. , Slavin, M. , Huang, H. Q. , Whent, M. , … Yu, L. L. (2012). Chemical composition of 13 commercial soybean samples and their antioxidant and anti‐inflammatory properties. Journal of Agricultural and Food Chemistry, 60(40), 10027–10034. 10.1021/jf303039a 22978480

