# Supplementary material for: Triacylglycerol, fatty acid, and phytochemical profiles in a new red sorghum variety (Ji Liang No. 1) and its antioxidant and anti‐inflammatory properties
Source: Food Sci Nutr. 2019 Feb 5;7(3):949–58. doi: 10.1002/fsn3.886 (PMC6418436; doi:10.1002/fsn3.886)
Supplement: Supplementary file 1 [file FSN3-7-949-s001.docx]

Supporting Information

**Triacylglycerol, fatty acid and phytochemical profiles in a new red sorghum variety (Ji Liang No.1) and its antioxidant and anti-inflammatory properties**

Yaqiong Zhang^1,2^, Ming Li^2^, Hang Gao^2^, Bo Wang^2^, Tongcheng Xu^3,^*, Boyan Gao^2,^*, Liangli (Lucy) Yu^4^

^1^Beijing Advanced Innovation Center for Food Nutrition and Human Health, Beijing Technology & Business University (BTBU), Beijing 100048, China

^2^Institute of Food and Nutraceutical Science, School of Agriculture and Biology, Shanghai Jiao Tong University, Shanghai 200240, China

^3^Institute of Agro-Food Science and Technology, Shandong Provincial Key Laboratory of agricultural products deep processing, Shandong Academy of Agricultural Science, Jinan 250100, China

^4^Department of Nutrition and Food Science, University of Maryland, College Park, MD 20742, United States

* To whom correspondence should be addressed:

Tongcheng Xu, Ph. D.

Tel.: 86-531-66659137; E-mail: xtc@live.com

Boyan Gao, Ph. D.

Tel.: 86-021-34204538; E-mail: [gaoboyan@sjtu.edu.cn](mailto:gaoboyan@sjtu.edu.cn)


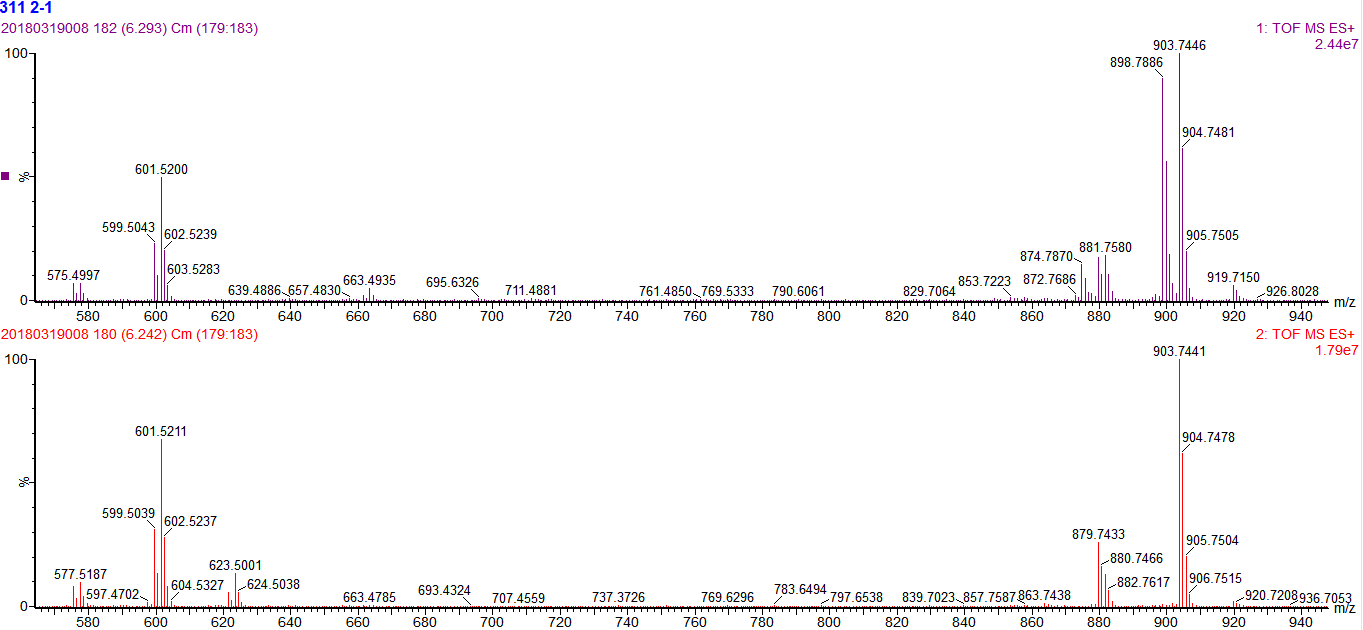


**FIGURE S1.** MS spectra of L-L-O (the peak 6). (Upper) MS^1^ spectrum, and (Lower) MS^2^ spectrum.


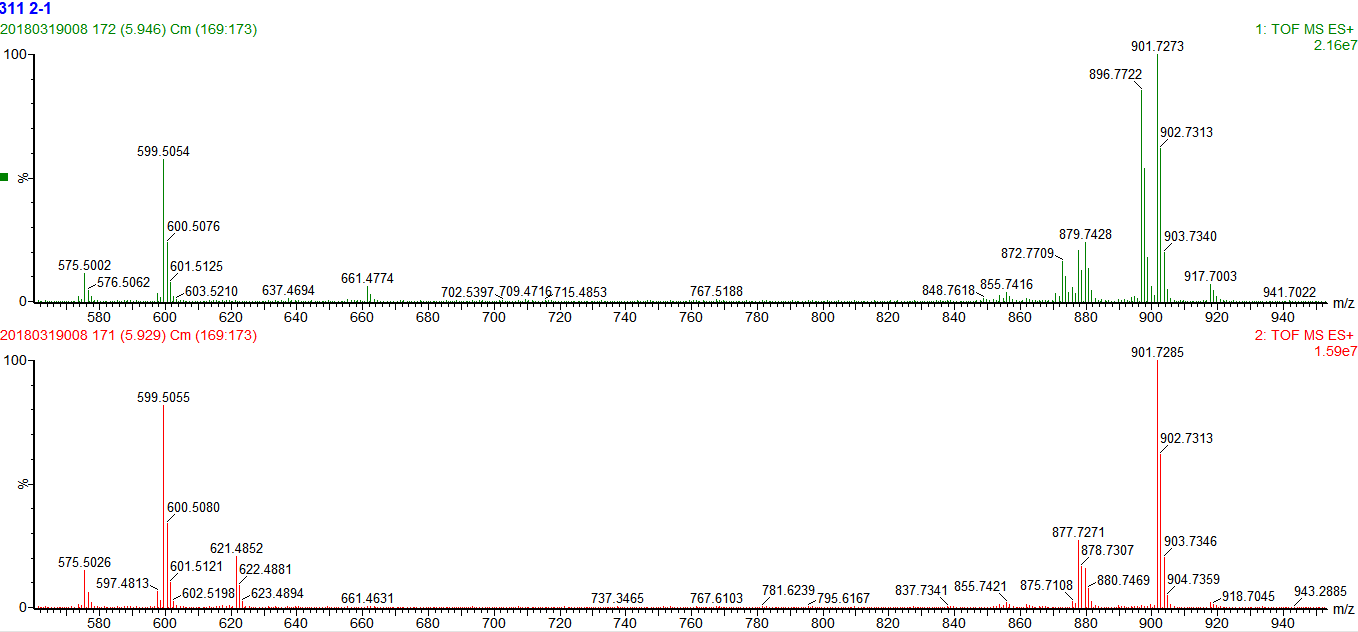


**FIGURE S2.** MS spectra of L-L-L (the peak 4). (Upper) MS^1^ spectrum, and (Lower) MS^2^ spectrum.


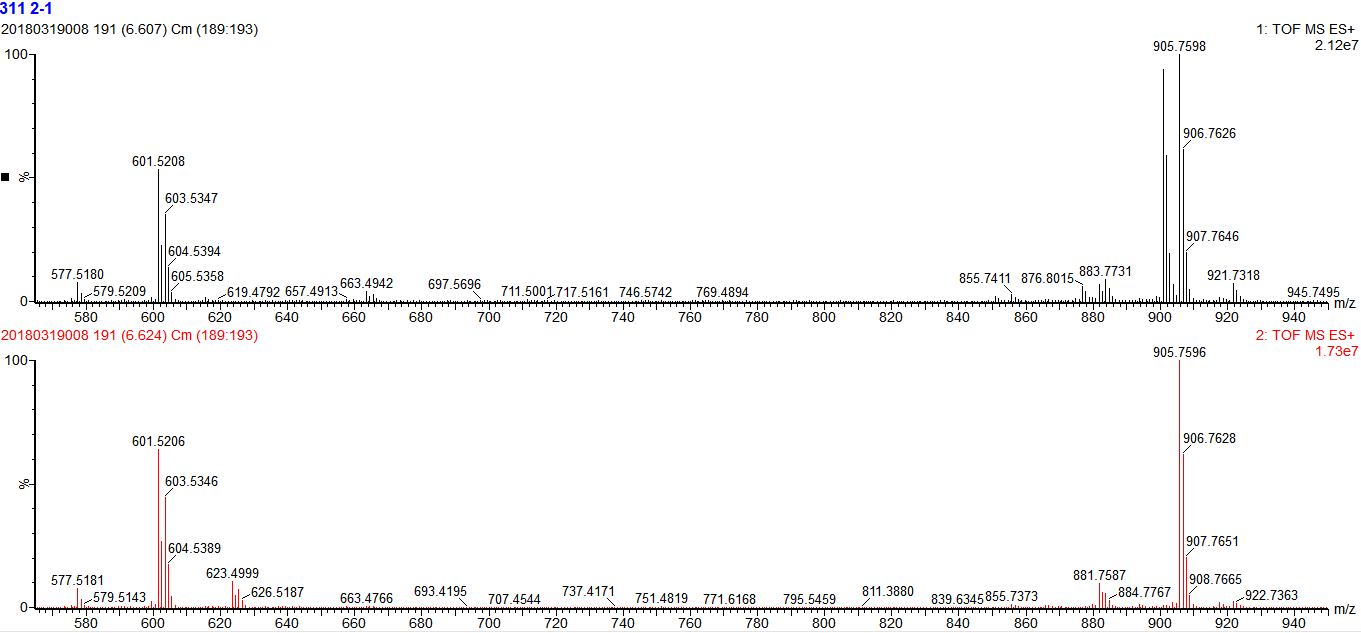


**FIGURE S3.** MS spectra of O-O-L (the peak 8). (Upper) MS^1^ spectrum, and (Lower) MS^2^ spectrum.
